# Completing a molecular timetree of Afrotheria

**DOI:** 10.3389/fbinf.2025.1710926

**Published:** 2025-11-19

**Authors:** Jack M. Craig, Whitney L. Fisher, Allan S. Thomas, S. Blair Hedges, Sudhir Kumar

**Affiliations:** 1 Institute for Genomics and Evolutionary Medicine, Temple University, Philadelphia, PA, United States; 2 Center for Biodiversity, Temple University, Philadelphia, PA, United States; 3 Department of Biology, Temple University, Philadelphia, PA, United States

**Keywords:** Afrotheria, KPg, phylogeny, dating, evolution

## Abstract

Afrotheria, the superorder that includes aardvarks, elephants, elephant shrews, hyraxes, manatees, and tenrecs, is home to some of the most charismatic and well-studied animals on Earth. Here, we assemble a nearly taxonomically complete molecular timetree of Afrotheria using an integrative approach that combines a literature search for published timetrees, *de novo* dating of untimed molecular phylogenies, and inference of timetrees from new alignments. The resulting timetree sheds light on the impact of the Cretaceous-Paleogene (K-Pg) role ∼66 million years ago in the diversification of Afrotherian orders. The earliest divergence in the timetree of Afrotherian mammals predates the K-Pg event by 12 million years, followed by five interordinal divergences that occurred gradually over a 16-million-year period encompassing the K-Pg event.

## Introduction

Six mammalian orders, containing some of the most charismatic species on Earth, such as elephants and manatees, share an evolutionary history that originated in Africa (Hedges, 2001). The elephants and mammoths (order Proboscidea), manatees and relatives (order Sirenia), aardvarks (order Tubulidentata), tenrecs and relatives (order Afrosoricida), hyraxes (order Hyracoidea), and elephant shrews (order Macroscelidea) together comprise Afrotheria, a clade supported by robust molecular phylogenies (Springer et al., 1997; Stanhope et al., 1998a; 1998b; Eizirik et al., 2001; Madsen et al., 2001; Murphy et al., 2001a; 2001b; van Dijk et al., 2001; Poulakakis and Stamatakis, 2010; Yazhini et al., 2021; Springer, 2022) and biogeographic data (Hedges, 2001; Wildman et al., 2007; Springer et al., 2011).

Africa was essentially an island from the end of the early Cretaceous to the mid-Eocene (105–40 mya) (Hedges, 2001), following its separation from South America and before establishing connections with Eurasia. During this time, Afrotherian mammals diversified to fill many ecological niches occupied by other orders of mammals on other continents (Gheerbrant et al., 2016; Grunstra et al., 2024) before gradually spreading outward (Sen, 2013). For example, golden moles (Afrosoricida) are anatomically and ecologically similar to true moles (Eulipotyphla: Talpidae), and elephant shrews (Macroscelidea) are similar to the nearly globally distributed shrews (Eulipotyphla: Soricidae).

However, while the phylogenetic and biogeographic signals are clear, the precise timing of the origins of all the members of Afrotherian orders remains unclear. The fossil record has been interpreted to suggest a 66 million-year divergence time between the majority of mammal orders, shortly after the Cretaceous–Paleogene (K-Pg) boundary, which marked the extinction of non-avian dinosaurs (Williamson et al., 2014). In this interpretation, the abundance of new mammal forms in the fossil record can be read to suggest an adaptive radiation into vacated niche space, which gave rise to modern mammal lineages, among them the Afrotherian orders (Meredith et al., 2011; O’Leary et al., 2013; Carlisle et al., 2023). However, molecular phylogenetic dating has converged on a median date of ∼90 mya for many mammalian superordinal divergences, which is 30 million years older than the oldest fossils would suggest (Hedges et al., 1996; Kumar and Hedges, 1998; Liu et al., 2017; Upham et al., 2021; Kumar et al., 2022; Foley et al., 2023). In light of these results, continental breakup has been invoked as the primary driver of major lineage divergences for placental mammals (Hedges et al., 1996; Foley et al., 2023).

Given their fundamental relationship to plate tectonics and geodispersal, the Afrotheria represent a compelling opportunity to test the hypothesis of radiation with a natural control for continental drift. Given that the six Afrotherian orders originated on an isolated continent, continental drift cannot have played a role at the ordinal level, and thus we have only to determine the timing of the major splits within Afrotheria to determine whether they occurred in rapid succession post-K-Pg, which would support the theory of rapid radiation, or whether there was a protracted period between the first and last ordinal split, with some divergences occurring before the K-Pg, which would fail to support this hypothesis. In other words, Afrotheria gives us a natural experiment to test whether the K-Pg impact can be invoked as a primary driver of diversification.

However, testing hypotheses on the basis of divergence times derived from incomplete phylogenies can lead to unreliable results (Wiens, 2003; Sanderson et al., 2010). Fortunately, it has been shown that nearly taxonomically complete phylogenies can often be assembled from existing published phylogenetic hypotheses without substantially acquiring new data (Craig et al., 2023a; Craig et al., 2024), thereby allowing for robust hypothesis testing without the need for resource-intensive new data collection. To build a nearly complete Afrotherian tree, we followed a three-step protocol (Craig et al., 2023a) resulting in a phylogeny of Afrotheria with 86 species, 100% of the extant species-level taxa recognized in the NCBI taxonomy database (Schoch et al., 2020). The NCBI taxonomy database only includes species that are in sequence databases. Our approach entailed: first, a literature search for timed molecular phylogenies; second, a search for molecular phylogenies with branch lengths proportional to genetic distance, for which we could impose chronological constraints; and finally, the assembly of novel sequence alignments from public data and *de novo* timetree construction. This protocol not only represents a flexible, resource-light approach that can be carried out for other taxa without the need for substantial new funding, field work, or laboratory work, but also a valuable way to leverage data that are otherwise trapped in static, published phylogenetic trees toward answering compelling evolutionary questions.

## Results

### The timetree of Afrotheria

The NCBI taxonomy database includes 86 species-level Afrotherian taxa with full binomial names. This number was obtained by excluding the following extinct members: four mammoths (*Mammuthus columbi*, *M. exilis*, *M. jeffersonii*, and *M. primigenius*), the North American mastodon (*Mammut americanum*), the Stellar’s sea cow *(Hydrodamalis gigas*), two elephants (*Elephas antiquus* and *E. cypriotes*), and the elephant relative *Notiomastodon platensis* (Schoch et al., 2020). Of these 86 species, 79 are present in the fifth edition of the TimeTree database (TT5) (Kumar et al., 2022). This leaves seven species for which molecular and possibly phylogenetic data exist; however, they have not been integrated into a comprehensive phylogenetic hypothesis: two golden moles (*Amblysomus robustus* and *Cryptochloris zyli*), the Benin tree hyrax (*Dendrohyrax interfluvialis*), the Karoo rock elephant shrew (*Elephantulus pilicaudus*), two additional elephant shrews (*Macroscelides flavicaudatus* and *M. micus*), and Dobson’s shrew tenrec (*Nesogale dobsoni*).

We conducted a multifaceted literature search for these seven missing Afrotherian species to find publications containing pertinent phylogenetic trees. We checked the source studies that contributed molecular data to GenBank for these species and then, if no tree was available, we searched Google Scholar for any timed molecular phylogenies that included them. Priority was given to phylogenies where divergence times were estimated by their respective authors (timetrees). This yielded five published phylogenies containing six species (Smit et al., 2011; Dumbacher, 2016; Bronner et al., 2024). Two of the phylogenies were timed (Murphy et al., 2007; Everson et al., 2016), and three were untimed (Smit et al., 2011; Dumbacher, 2016; Bronner et al., 2024). This left only the Benin tree hyrax (*Dendrohyrax interfluvialis*) unrepresented.

For *Dendrohyrax interfluvialis*, we accessed a sequence for cytochrome c oxidase subunit I (CO1), which is commonly used for species identification and phylogenetics, from GenBank (QZR93760.1) and subjected it to an NCBI GCR smartBLAST analysis, which identified GenBank accessions for homologous markers from related species and outgroup taxa (the species lists are included in the Methodological Details). We visually inspected the alignment in MEGA (Tamura et al., 2021) and then built a phylogeny in MEGA and timed it using RelTime (Tamura et al., 2012). The accession list, alignment file, and intermediary tree files are available in the Supplementary Material.

In the case of every published tree we found, we observed that the final Newick tree files were not made available with their respective publications, making it difficult to integrate these phylogenetic hypotheses with downstream macroevolutionary projects such as ours. We manually created Newick tree files by faithfully tracing the figures presented in each manuscript, thereby preserving the topology and branch lengths (timed or untimed) estimated by the original authors. We did so by capturing the topology in the manual tree editor in MEGA 12 (Tamura et al., 2021) and carefully determined the branch lengths using ImageJ (Schneider et al., 2012). We then visually inspected each of our Newick trees and corrected any discrepancies with the source by manually editing the resulting Newick string in a rich text editor to ensure accurate reproduction of the published tree figure. This step is essential to resurrect valuable contributions that are essentially frozen out of modern, bioinformatic-scale evolutionary studies. These tree files are available in the Supplementary Material.

For the three phylogenies that had not been timed by their original authors (Smit et al., 2011; Dumbacher, 2016; Bronner et al., 2024), we imposed literature-consensus calibrations in the RelTime analysis (Tamura et al., 2012; Tamura et al., 2018) following our secondary calibration protocol (Craig et al., 2023a; Craig et al., 2024) in order to convert the branch lengths from units of genetic distance to absolute time. The timed tree files are available in the Supplementary Material. This protocol benefits from using the corpus of divergence time estimates synthesized by the TimeTree of Life database to constrain divergences that would otherwise lack pertinent or unambiguous fossil calibrations (Craig et al., 2023a; Craig et al., 2024).

**FIGURE 1:**
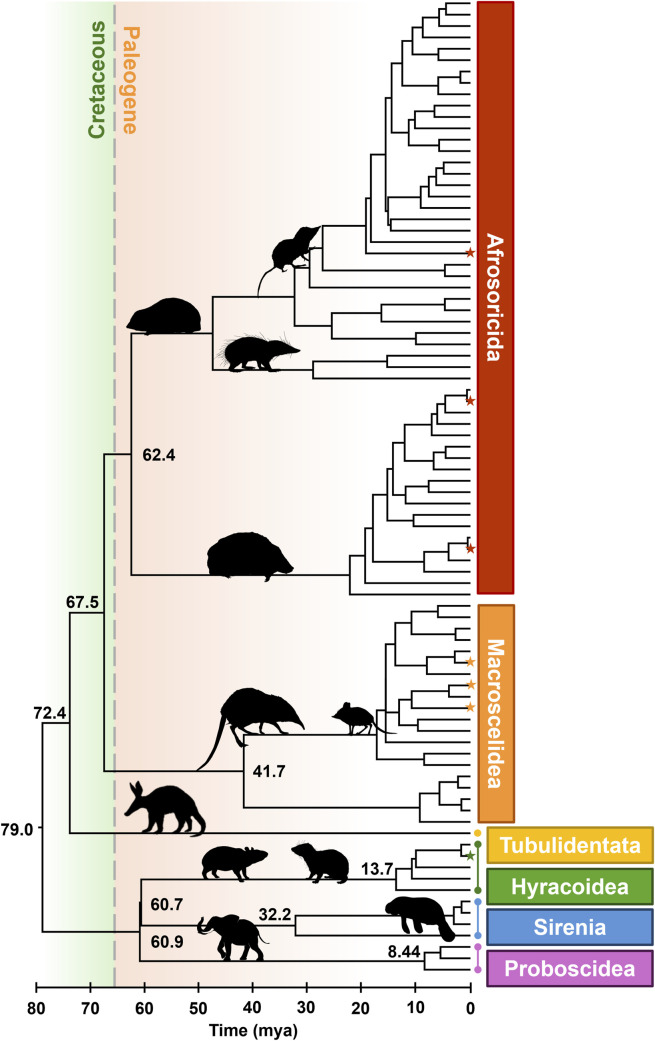
Phylogeny of 86 extant Afrotherian species, representing six orders. The K-Pg border is shown with a dashed line. Stars indicate species newly added to the tree. All times in millions of years ago (mya). Images from Phylopic.org.

**FIGURE 2:**
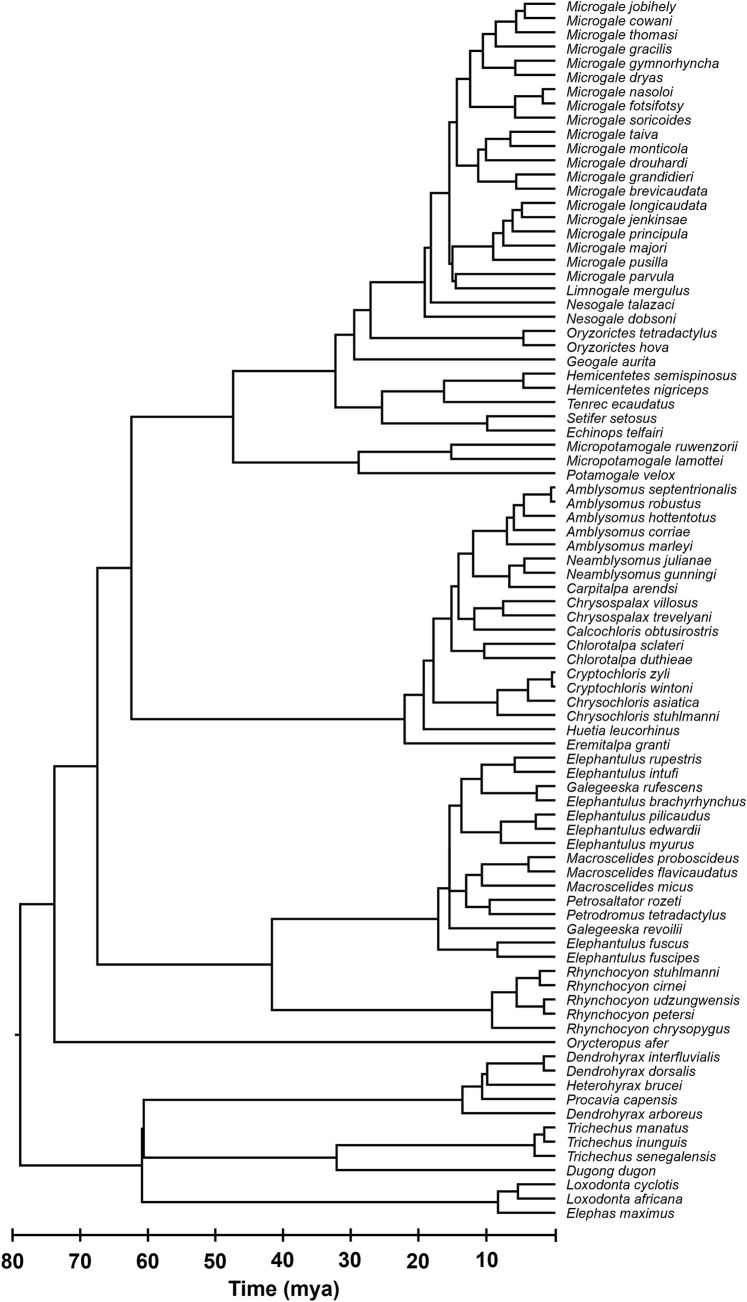
Phylogeny of 86 extant Afrotherian species, representing six orders. All species names are shown for clarity.

Finally, we used Chrono-STA (Barba-Montoya et al., 2025) to synthesize all the timetrees, including the TT5 backbone, published timetrees (Murphy et al., 2007; Everson et al., 2016), three newly timed phylogenies (Smit et al., 2011; Dumbacher, 2016; Bronner et al., 2024), and our novel timetree for *Dendrohyrax interfluvialis*. This yielded a final super-timetree of Afrotheria, in which every tip and node age is informed by molecular data (Figure 1). It incorporates 86 species (Figure 2), representing 100% of all extant Afrotherians in the NCBI taxonomic database.

We observed the crown age of Afrotheria to be 79.0 mya, which is approximately 12 million years prior to the K-Pg boundary (66 mya) (Figure 1). Similarly, the split between Tubulidentata and the clade comprising Afrosoricida and Macroscelidea occurred at 72.4 mya, and the split between Afrosoricida and Macroscelidea occurred at 67.5 mya. The splits between the remaining three orders are younger than the K-Pg. We estimated that Hyracoidea and Sirenia split 60.7 mya, and these two split from Proboscidea 60.9 mya, while Afrosoricida split from Macroscelidea 62.4 mya. In other words, the deepest splits within Afrotheria occurred prior to the K-Pg event, while the younger half of the ordinal splits occurred shortly thereafter. This represents a span of 18.3 million years from the beginning to the end of Afrotherian ordinal diversification.

## Discussion

We assembled a molecular phylogeny of 86 Afrotherian species through the synthesis of published timed phylogenies, untimed phylogenies, and molecular sequences. Such large-scale, nearly complete phylogenies are still relatively rare in the field, even for exceptionally well-studied groups (Barba-Montoya et al., 2025), but they are highly valuable for downstream work in evolutionary biology and conservation.

For example, this new tree allowed us to assess the impact of the K-Pg event on ordinal diversification within Afrotheria. We found that the basal split occurred at 79.0 mya, prior to the K-Pg, which is consistent with several other pre-K-Pg estimates (Nikaido et al., 2001; Arnason and Janke, 2002; Douady and Douzery, 2003; Hasegawa et al., 2003; Murphy et al., 2007; Hallström and Janke, 2008; 2010; Morrison, 2009; Meredith et al., 2011; Soares et al., 2013). Similarly, the second split, between aardvarks (Tubulidentata) and the clade comprised of tenrecs (Afrosoricida) and elephant shrews (Macroscelidea), occurred at 72.4 mya, which is consistent with several previously reported dates (Douady and Douzery, 2003; Murphy et al., 2007; Hedges and Kumar, 2009). Furthermore, the subsequent divergence between tenrecs and elephant shrews occurred at 67.5 mya in our tree, which is also consistent with prior work (Douady and Douzery, 2003; Kitazoe et al., 2007). Crucially, Afrotherian ordinal splits span 18.3 million years from the first to the last intra-ordinal divergence, or approximately 20% of the total age of the clade. Thus, the ordinal divergences within Afrotheria began prior to the K-Pg event and spanned a substantial portion of the history of the clade, which cannot be interpreted as evidence supporting a rapid, post-K-Pg radiation of Afrotherian orders.

In contrast, some prior studies found a post-K-Pg timing for the basal Afrotherian divergence, for example, Heritage and Seiffert (2022) at 65.4 mya and Laurin et al. (2022) at 64.9 mya. This is likely attributable to the use of restrictive time calibrations (Hedges et al., 2018). Heritage and Seiffert (2022) imposed 16 calibrations on a tree of only 65 taxa, including a crown-Afrotherian calibration centered at 60 mya. Laurin et al. (2022) imposed a broad set of fossil calibrations drawn from 25 separate publications on a tree with 95 taxa, but their position and ranges are not explicitly stated. In other words, both studies temporally constrained at least a quarter of the internal nodes in their trees. Importantly, these two cases reflect a minority position, as the other 22 studies in the TimeTree database estimated the crown-Afrotherian divergence to have occurred 70 mya or earlier, which is decisively pre-K-Pg. Thus, the present work demonstrates the value of large-scale syntheses in determining consensus timing for major divergences across the tree of life.

Finally, the tree we assembled here represents a useful synthesis of decades of work in Afrotherian phylogenetics. We hope it can serve as a blueprint for future large-scale synthetic molecular trees of other well-studied groups.

## Methodological details

### Taxonomic reference

We used the NCBI taxonomy database (Schoch et al., 2020) as a taxonomic framework. We identified 86 binomial taxa, excluding all extinct species (e.g., mammoths), taxa of uncertain taxonomic identity (indicated by an ICZN abbreviation, such as “sp.”), hybrids, redundant subspecies (retaining the type *ssp*. if no binomial name is recognized), and regional variants. After this careful curation, our target species list for literature searches included 86 Afrotherians.

It should be noted that some minor taxonomic disagreements exist in Afrotherian taxonomy. The International Union for Conservation of Nature (IUCN) Red List recognizes a total of 88 Afrotherian species compared to 86 in the NCBI. *Calcochloris tytonis*, *Chrysochloris visagiei*, *Dendrohyrax validus*, and *Hydrodamalis gigas* are present in the IUCN Red List but not in the NCBI, while *Dendrohyrax int*erfluvialis and *Rhynchocyon stuhlmanni* are present in the NCBI but not in the IUCN. Furthermore, the NCBI has the names *Huetia leucorhinus* and *Limnogale mergulus,* while the IUCN Red List uses the synonyms *Huetia leucorhina* and *Microgale mergulus*. It is outside the scope of this article to carry out a full taxonomic revision of these clades. Because renaming several tip taxa would have little bearing on our final phylogenetic hypothesis, we will defer to the NCBI throughout.

### Phylogeny processing

We captured the phylogenies as Newick strings from published image files of five studies by manually constructing the topology in MEGA version 12 (Tamura et al., 2021), then measuring and reproducing branch lengths in ImageJ 1.53k (Schneider et al., 2012). Branch lengths were converted from measured lengths to pixels to either millions of years for timed trees or molecular substitutions for untimed trees using the provided scale bars. To determine if we could accurately reproduce a phylogeny as a Newick file from an image embedded in a publication PDF, we first generated an example tree, saved it as a 300 DPI JPEG image, and created a Newick file 10 times, then observed the range of times produced for each node. We found that on average, node heights were estimated within approximately 1 million years of the true times we used to build the initial tree. We deemed this minor degree of imprecision sufficient for our purposes in distance-based phylogeny synthesis, while acknowledging that any uncertainty in time estimates reported in the associated publications was always greater. These trees are present in the Supplementary Material as “raw trees.”

In cases where figures in publications contained tips with labels that did not appear in the NCBI taxonomic reference, these tips were pruned prior to super-timetree inference. Such discrepancies typically stem from taxonomic discord. For example, Bronner et al. (2024) included the tip label “*Kilimatalpa stuhlmanni*,” whereas the NCBI taxonomy lacks this name but did have “*Chrysochloris stuhlmanni*.” In cases like this, identifying synonymy and merging the two names is simple, but in others, it would require a degree of domain-specific taxonomic expertise that is outside the scope of the present work. Rather than attempting to reconcile the names present in every study with both the NCBI taxonomy and the TimeTree database, we maintained the NCBI as our sole reference, simply dropping any tips that did not appear therein. Furthermore, we removed tips with redundant labels (*e.g.*, multiple members of the same species), as these were unnecessary for our primary goal of integrating new species into the TimeTree backbone. Similarly, if a study tree had multiple species that were already present in the backbone, these could be dropped, as the Chrono-STA approach used for super-timetree construction was developed specifically for merging timetrees on the basis of minimal taxonomic overlap. In fact, only a single tip needs to be shared between a study tree and the backbone for them to be merged seamlessly; therefore, pruning redundant tips in the study trees did not impact our ability to integrate the new species.

Similarly, when study trees contained tips that were not resolved to the species level [for example, Everson et al. (2016), who used single tips labeled “Elephant” and “Hyrax” in their tree as placeholders for their respective clades], we used species-level representative taxa (*Elephas maximus* and *Dendrohyrax dorsalis*) in their place. This was possible because, by phylogenetic first principles, any two members of a single clade are equidistant from their sister clade. In other words, all elephants share the same divergence time from all hyraxes or any other Afrotherian. Using a representative elephant here facilitates super-timetree construction with Chrono-STA by increasing taxonomic overlap, but, crucially, unlike the case with renamed taxa, this does not risk any taxonomic confusion because all members of the given group are phylogenetically equal in this context. This becomes especially clear when considering that Chrono-STA is a distance-based method that is wholly agnostic of topology.

Thus, where differences between the published versions of the trees and the ones we used in our analysis do exist, typically in the form of taxa that were dropped, and in a few cases due to the choice of representative taxa used to replace compressed clades, we made these modifications to facilitate super-timetree inference and minimize any potential bias induced due to taxonomic discord. Crucially, because we used a super-timetree approach developed specifically for reconciling sparsely sampled timetrees, we can be sure that taking the conservative step of pruning contested or redundant tips does not negatively impact our final super-timetree. Pruned trees and those with representative taxa are present in the Supplementary Material as “final trees,” and these were the exact files used in super-timetree inference.

We timed the three untimed trees using a literature consensus secondary calibration approach developed in previous work (Craig et al., 2023a; Craig et al., 2023b). We constructed a relative timetree using RelTime (Tamura et al., 2012; Tamura et al., 2018) in MEGA (Kumar et al., 2024), then selected a basal divergence to calibrate and estimated divergence times for all ingroup species. We accessed a distribution of published divergence times from the TimeTree database in each case. We used TT5’s reported 95% CI of published time estimates to define a uniform confidence interval imposed on the selected node. These newly-timed phylogenies are also present in the Supplementary Material as “final trees”, whereas their untimed equivalents are “raw trees.”

### Novel phylogeny building

We found that *Dendrohyrax interfluvialis* had available molecular data in GenBank, but we did not find a published molecular phylogeny that contained this taxon. Therefore, we searched GenBank accessions for *D. interfluvialis* to identify any record of a mitochondrial protein greater than 100 amino acids in length. This would form the basis of a simple molecular phylogeny from which we could extract relevant divergence times.

We selected a mitochondrial cytochrome c oxidase subunit I protein sequence (QZR93760.1) and submitted it to NIH CGR SmartBLAST to find protein homologs. We then selected several closely related members of Hyracoidea as ingroup taxa (*Crossarchus obscurus*, *Dendrohyrax dorsalis*, *Heterohyrax brucei*, and *Procavia capensis*), plus several reference organisms as outgroups (*Mus musculus*, *Homo sapiens*, *Danio rerio*, *Drosophila melanogaster*, and *Caenorhabditis elegans*). We exported these sequences to a FASTA file using GenPept, aligned and trimmed excess sequences from the ends where necessary, and built a molecular phylogeny in MEGA (Tamura et al., 2021) using a maximum likelihood search via nearest-neighbor interchange (NNI) for each under the JTT model. We used adaptive bootstraps (Sharma and Kumar, 2021) with an adaptive parameter search to test the confidence of our topology. We then timed this tree in RelTime (Tamura et al., 2012; Tamura et al., 2018) as described above. The accession list, alignment, and tree files are available in the Supplementary Material.

### Chrono-STA

To combine all timetrees, we ran the supertree unification algorithm Chrono-STA (Barba-Montoya et al., 2025). We used the first revised weighted approach implemented in Chrono-STA, which is publicly available on their GitHub repository: https://github.com/josebarbamontoya/chrono-sta. The final phylogeny, with all tip labels shown for clarity, can be seen in Figure 2. Importantly, Chrono-STA was developed specifically to overcome sparse taxonomic sampling and high variance among different time estimates of the same phylogenetic divergence on the basis of the shared chronological scale. Phylogenies are converted to patristic pairwise time matrices, rendering the method topology-agnostic. Then, a modified recursive UPGMA algorithm is applied to integrate multiple small phylogenies into a single, comprehensive distance matrix with no gaps. For this reason, including redundant tips, such as those present in the TT5 backbone, is superfluous. This allowed us to prune them as needed to minimize error stemming from taxonomic flux.

To capture the charismatic extinct Proboscidean lineages (mammoths and mastodons), we subsequently accessed a phylogeny of Proboscidea from TT5, which included extinct taxa supported by the publication Heritage and Seiffert (2022). We then ran Chrono-STA to merge our consensus phylogeny with this Proboscidean phylogeny, resulting in a tree with the additional species *Paleoloxodon antiquus, Mammuthus primigenius, Mammut americanum,* and *Losodokodon losodokinus*. These are of great interest to some researchers, but as extinct species, they fall outside the scope of this project. We included the phylogeny of these species in the Supplementary Material.

## Data Availability

The original contributions presented in the study are included in the article/Supplementary Material; further inquiries can be directed to the corresponding author.

## References

[B1] Arnason U. Janke A. (2002). Mitogenomic analyses of eutherian relationships. Cytogenet. Genome Res. 96, 20–32. 10.1159/000063023 12438776

[B2] Barba-Montoya J. Craig J. M. Kumar S. (2025). Integrating phylogenies with chronology to assemble the tree of life. Front. Bioinform 5, 1571568. 10.3389/fbinf.2025.1571568 40370941 PMC12075222

[B3] Bronner G. N. Mynhardt S. Bennett N. C. Cohen L. Crumpton N. Hofreiter M. (2024). Phylogenetic history of golden moles and tenrecs (Mammalia: Afrotheria). Zool. J. Linn. Soc. 201, 184–213. 10.1093/zoolinnean/zlad121

[B4] Carlisle E. Janis C. M. Pisani D. Donoghue P. C. J. Silvestro D. (2023). A timescale for placental mammal diversification based on Bayesian modeling of the fossil record. Curr. Biol. 33, 3073–3082.e3. 10.1016/j.cub.2023.06.016 37379845 PMC7617171

[B5] Craig J. M. Bamba G. L. Barba-Montoya J. Hedges S. B. Kumar S. (2023a). Completing a molecular timetree of apes and monkeys. Front. Bioinform 3, 1284744. 10.3389/fbinf.2023.1284744 38162123 PMC10757846

[B6] Craig J. M. Kumar S. Hedges S. B. (2023b). The origin of eukaryotes and rise in complexity were synchronous with the rise in oxygen. Front. Bioinforma. 3, 1233281. 10.3389/fbinf.2023.1233281 37727796 PMC10505794

[B7] Craig J. M. Hedges S. B. Kumar S. (2024). Completing a molecular timetree of primates. Front. Bioinform 4, 1495417. 10.3389/fbinf.2024.1495417 39737248 PMC11683086

[B8] Douady C. J. Douzery E. J. P. (2003). Molecular estimation of eulipotyphlan divergence times and the evolution of “Insectivora.”. Mol. Phylogenet. Evol. 28, 285–296. 10.1016/s1055-7903(03)00119-2 12878465

[B9] Dumbacher J. P. (2016). Petrosaltator gen. nov., a new genus replacement for the North African sengi Elephantulus rozeti (Macroscelidea; Macroscelididae). Zootaxa 4136, 567–579. 10.11646/zootaxa.4136.3.8 27395734

[B10] Eizirik E. Murphy W. J. O’Brien S. J. (2001). Molecular dating and biogeography of the early placental mammal radiation. J. Hered. 92, 212–219. 10.1093/jhered/92.2.212 11396581

[B11] Everson K. M. Soarimalala V. Goodman S. M. Olson L. E. (2016). Multiple loci and complete taxonomic sampling resolve the phylogeny and biogeographic history of tenrecs (mammalia: tenrecidae) and reveal higher speciation rates in Madagascar’s humid forests. Syst. Biol. 65, 890–909. 10.1093/sysbio/syw034 27103169

[B12] Foley N. M. Mason V. C. Harris A. J. Bredemeyer K. R. Damas J. Lewin H. A. (2023). A genomic timescale for placental mammal evolution. Science 380, eabl8189. 10.1126/science.abl8189 37104581 PMC10233747

[B13] Gheerbrant E. Filippo A. Schmitt A. (2016). Convergence of afrotherian and laurasiatherian ungulate-like mammals: first morphological evidence from the Paleocene of Morocco. PLoS One 11, e0157556. 10.1371/journal.pone.0157556 27384169 PMC4934866

[B14] Grunstra N. D. S. Hollinetz F. Bravo Morante G. Zachos F. E. Pfaff C. Winkler V. (2024). Convergent evolution in Afrotheria and Non-afrotherians demonstrates high evolvability of the mammalian inner ear. Nat. Commun. 15, 7869. 10.1038/s41467-024-52180-1 39285191 PMC11405882

[B15] Hallström B. M. Janke A. (2008). Resolution among major placental mammal interordinal relationships with genome data imply that speciation influenced their earliest radiations. BMC Evol. Biol. 8, 162. 10.1186/1471-2148-8-162 18505555 PMC2435553

[B16] Hallström B. M. Janke A. (2010). Mammalian evolution may not be strictly bifurcating. Mol. Biol. Evol. 27, 2804–2816. 10.1093/molbev/msq166 20591845 PMC2981514

[B17] Hasegawa M. Thorne J. L. Kishino H. (2003). Time scale of eutherian evolution estimated without assuming a constant rate of molecular evolution. Genes Genet. Syst. 78, 267–283. 10.1266/ggs.78.267 14532706

[B18] Hedges S. B. (2001). Afrotheria: plate tectonics meets genomics. Proc. Natl. Acad. Sci. U. S. A. 98, 1–2. 10.1073/pnas.98.1.1 11136239 PMC33345

[B19] Hedges S. B. Kumar S. (2009). The timetree of life. Oxford, UK: Oxford University Press.

[B20] Hedges S. B. Parker P. H. Sibley C. G. Kumar S. (1996). Continental breakup and the ordinal diversification of birds and mammals. Nature 381, 226–229. 10.1038/381226a0 8622763

[B21] Hedges S. B. Tao Q. Walker M. Kumar S. (2018). Accurate timetrees require accurate calibrations. Proc. Natl. Acad. Sci. U. S. A. 115, E9510–E9511. 10.1073/pnas.1812558115 30266795 PMC6187123

[B22] Heritage S. Seiffert E. R. (2022). Total evidence time-scaled phylogenetic and biogeographic models for the evolution of sea cows (Sirenia, Afrotheria). PeerJ 10, e13886. 10.7717/peerj.13886 36042864 PMC9420408

[B23] Kitazoe Y. Kishino H. Waddell P. J. Nakajima N. Okabayashi T. Watabe T. (2007). Robust time estimation reconciles views of the antiquity of placental mammals. PLoS One 2, e384. 10.1371/journal.pone.0000384 17440620 PMC1849890

[B24] Kumar S. Hedges S. B. (1998). A molecular timescale for vertebrate evolution. Nature 268, 917–920. 10.1038/31927 9582070

[B25] Kumar S. Suleski M. Craig J. M. Kasprowicz A. E. Sanderford M. Li M. (2022). TimeTree 5: an expanded resource for species divergence times. Mol. Biol. Evol. 39, msac174. 10.1093/molbev/msac174 35932227 PMC9400175

[B26] Kumar S. Stecher G. Suleski M. Sanderford M. Sharma S. Tamura K. (2024). MEGA12: molecular evolutionary genetic analysis version 12 for adaptive and green computing. Mol. Biol. Evol. 41, msae263. 10.1093/molbev/msae263 39708372 PMC11683415

[B27] Laurin M. Lapauze O. Marjanović D. (2022). What do ossification sequences tell us about the origin of extant amphibians? Peer Community J. 2, e12. 10.24072/pcjournal.89

[B28] Liu L. Zhang J. Rheindt F. E. Lei F. Qu Y. Wang Y. (2017). Genomic evidence reveals a radiation of placental mammals uninterrupted by the KPg boundary. Proc. Natl. Acad. Sci. U. S. A. 114, E7282–E7290. 10.1073/pnas.1616744114 28808022 PMC5584403

[B29] Madsen O. Scally M. Douady C. J. Kao D. J. DeBry R. W. Adkins R. (2001). Parallel adaptive radiations in two major clades of placental mammals. Nature 409, 610–614. 10.1038/35054544 11214318

[B30] Meredith R. W. Janečka J. E. Gatesy J. Ryder O. A. Fisher C. A. Teeling E. C. (2011). Impacts of the Cretaceous Terrestrial revolution and KPg extinction on mammal diversification. Science 334, 521–524. 10.1126/science.1211028 21940861

[B31] Morrison D. A. (2009). The timetree of life. In Hedges S. B. Kumar S. , editors. Systematic biology. Oxford, Uk: Oxford University Press. p. 461–462.

[B32] Murphy W. J. Eizirik E. Johnson W. E. Zhang Y. P. Ryder O. A. O’Brien S. J. (2001a). Molecular phylogenetics and the origins of placental mammals. Nature 409, 614–618. 10.1038/35054550 11214319

[B33] Murphy W. J. Eizirik E. O’Brien S. J. Madsen O. Scally M. Douady C. J. (2001b). Resolution of the early placental mammal radiation using Bayesian phylogenetics. Science 294, 2348–2351. 10.1126/science.1067179 11743200

[B34] Murphy W. J. Pringle T. H. Crider T. A. Springer M. S. Miller W. (2007). Using genomic data to unravel the root of the placental mammal phylogeny. Genome Res. 17, 413–421. 10.1101/gr.5918807 17322288 PMC1832088

[B35] Nikaido M. Kawai K. Cao Y. Harada M. Tomita S. Okada N. (2001). Maximum likelihood analysis of the complete mitochondrial genomes of eutherians and a reevaluation of the phylogeny of bats and insectivores. J. Mol. Evol. 53, 508–516. 10.1007/s002390010241 11675611

[B36] O’Leary M. A. Bloch J. I. Flynn J. J. Gaudin T. J. Giallombardo A. Giannini N. P. (2013). The placental mammal ancestor and the post-K-Pg radiation of placentals. Science 339, 662–667. 10.1126/science.1229237 23393258

[B37] Poulakakis N. Stamatakis A. (2010). Recapitulating the evolution of Afrotheria: 57 genes and rare genomic changes (RGCs) consolidate their history. Syst. Biodivers. 8, 395–408. 10.1080/14772000.2010.484436

[B38] Sanderson M. J. McMahon M. M. Steel M. (2010). Phylogenomics with incomplete taxon coverage: the limits to inference. BMC Evol. Biol. 10, 155. 10.1186/1471-2148-10-155 20500873 PMC2897806

[B39] Schneider C. A. Rasband W. S. Eliceiri K. W. (2012). NIH Image to ImageJ: 25 years of image analysis. Nat. Methods 9, 671–675. 10.1038/nmeth.2089 22930834 PMC5554542

[B40] Schoch C. L. Ciufo S. Domrachev M. Hotton C. L. Kannan S. Khovanskaya R. (2020). NCBI taxonomy: a comprehensive update on curation, resources and tools. Database 2020, baaa062–21. 10.1093/database/baaa062 32761142 PMC7408187

[B41] Sen S. (2013). Dispersal of African mammals in eurasia during the Cenozoic: ways and whys. Geobios 46, 159–172. 10.1016/j.geobios.2012.10.012

[B42] Sharma S. Kumar S. (2021). Fast and accurate bootstrap confidence limits on genome-scale phylogenies using little bootstraps. Nat. Comput. Sci. 1, 573–577. 10.1038/s43588-021-00129-5 34734192 PMC8560003

[B43] Smit H. A. Jansen van Vuuren B. O’Brien P. C. M. Ferguson-Smith M. Yang F. Robinson T. J. (2011). Phylogenetic relationships of elephant‐shrews (Afrotheria, Macroscelididae): Elephant-shrew phylogeny. J. Zool. (1987) 284, 133–143. 10.1111/j.1469-7998.2011.00790.x

[B44] Soares P. Abrantes D. Rito T. Thomson N. Radivojac P. Li B. (2013). Evaluating purifying selection in the mitochondrial DNA of various mammalian species. PLoS One 8, e58993. 10.1371/journal.pone.0058993 23533597 PMC3606437

[B45] Springer M. S. (2022). Afrotheria. Curr. Biol. 32, R205–R210. 10.1016/j.cub.2022.02.001 35290765

[B46] Springer M. S. Cleven G. C. Madsen O. de Jong W. W. Waddell V. G. Amrine H. M. (1997). Endemic African mammals shake the phylogenetic tree. Nature 388, 61–64. 10.1038/40386 9214502

[B47] Springer M. S. Meredith R. W. Janecka J. E. Murphy W. J. (2011). The historical biogeography of Mammalia. Philos. Trans. R. Soc. Lond. B Biol. Sci. 366, 2478–2502. 10.1098/rstb.2011.0023 21807730 PMC3138613

[B48] Stanhope M. J. Madsen O. Waddell V. G. Cleven G. C. de Jong W. W. Springer M. S. (1998a). Highly congruent molecular support for a diverse superordinal clade of endemic African mammals. Mol. Phylogenet Evol. 9, 501–508. 10.1006/mpev.1998.0517 9667998

[B49] Stanhope M. J. Waddell V. G. Madsen O. de Jong W. Hedges S. B. Cleven G. C. (1998b). Molecular evidence for multiple origins of insectivora and for a new order of endemic African insectivore mammals. Proc. Natl. Acad. Sci. U. S. A. 95, 9967–9972. 10.1073/pnas.95.17.9967 9707584 PMC21445

[B50] Tamura K. Battistuzzi F. U. Billing-Ross P. Murillo O. Filipski A. Kumar S. (2012). Estimating divergence times in large molecular phylogenies. Proc. Natl. Acad. Sci. U. S. A. 109, 19333–19338. 10.1073/pnas.1213199109 23129628 PMC3511068

[B51] Tamura K. Tao Q. Kumar S. (2018). Theoretical foundation of the reltime method for estimating divergence times from variable evolutionary rates. Mol. Biol. Evol. 35, 1770–1782. 10.1093/molbev/msy044 29893954 PMC5995221

[B52] Tamura K. Stecher G. Kumar S. (2021). MEGA11: molecular evolutionary genetics analysis version 11. Mol. Biol. Evol. 38, 3022–3027. 10.1093/molbev/msab120 33892491 PMC8233496

[B53] Upham N. S. Esselstyn J. A. Jetz W. (2021). Molecules and fossils tell distinct yet complementary stories of mammal diversification. Curr. Biol. 31, 4195–4206.e3. 10.1016/j.cub.2021.07.012 34329589 PMC9090300

[B54] van Dijk M. A. Madsen O. Catzeflis F. Stanhope M. J. de Jong W. W. Pagel M. (2001). Protein sequence signatures support the African clade of mammals. Proc. Natl. Acad. Sci. U. S. A. 98, 188–193. 10.1073/pnas.98.1.188 11114173 PMC14566

[B55] Wiens J. J. (2003). Missing data, incomplete taxa, and phylogenetic accuracy. Syst. Biol. 52, 528–538. 10.1080/10635150309308 12857643

[B56] Wildman D. E. Uddin M. Opazo J. C. Liu G. Lefort V. Guindon S. (2007). Genomics, biogeography, and the diversification of placental mammals. Proc. Natl. Acad. Sci. U. S. A. 104, 14395–14400. 10.1073/pnas.0704342104 17728403 PMC1958817

[B57] Williamson T. E. Brusatte S. L. Wilson G. P. (2014). The origin and early evolution of metatherian mammals: the Cretaceous record. Zookeys 465, 1–76. 10.3897/zookeys.465.8178 25589872 PMC4284630

[B58] Yazhini A. Srinivasan N. Sandhya S. (2021). Signatures of conserved and unique molecular features in Afrotheria. Sci. Rep. 11, 1011. 10.1038/s41598-020-79559-6 33441654 PMC7806701

